# An In-Hospital Mortality Prediction Model for Acute Pesticide Poisoning in the Emergency Department

**DOI:** 10.3390/toxics13100893

**Published:** 2025-10-18

**Authors:** Yoonseo Jeon, Da-Eun Kim, Inyong Jeong, Se-Jin Ahn, Nam-Jun Cho, Hyo-Wook Gil, Hwamin Lee

**Affiliations:** 1 College of Medicine, Korea University, Seoul 02841, Republic of Korea; 2 Department of Biomedical Informatics, Korea University College of Medicine, Seoul 02841, Republic of Korea; 3 Department of Internal Medicine, Soonchunhyang University Cheonan Hospital, Cheonan 31151, Republic of Korea

**Keywords:** acute pesticide poisoning, in-hospital mortality, emergency department, machine learning, logistic regression

## Abstract

Pesticide poisoning remains a significant public health issue, characterized by high morbidity and mortality, particularly among patients presenting to the emergency department. This study aimed to develop a 14-day in-hospital mortality prediction model for patients with acute pesticide poisoning using early clinical and laboratory data. This retrospective cohort study included 1056 patients who visited Soonchunhyang University Cheonan Hospital between January 2015 and December 2020. The cohort was randomly divided into train (*n* = 739) and test (*n* = 317) sets using stratification by pesticide type and outcome. Candidate predictors were selected based on univariate Cox regression, LASSO regularization, random forest feature importance, and clinical relevance derived from established prognostic scoring systems. Logistic regression models were constructed using six distinct feature sets. The best-performing model combined LASSO-selected and clinically curated features (AUC 0.926 [0.890–0.957]), while the final model—selected for interpretability—used only LASSO-selected features (AUC 0.923 [0.884–0.955]; balanced accuracy 0.835; sensitivity 0.843; specificity 0.857; F1.5 score 0.714 at threshold 0.450). SHapley Additive exPlanations (SHAP) analysis identified paraquat ingestion, Glasgow Coma Scale, bicarbonate level, base excess, and alcohol history as major mortality predictors. The proposed model outperformed the APACHE II score (AUC 0.835 [0.781–0.888]) and may serve as a valuable tool for early risk stratification and clinical decision making in pesticide-poisoned patients.

## 1. Introduction

Pesticide ingestion remains a significant public health issue, particularly in many Asian countries, where it is a common method of suicide and a leading cause of toxic exposure requiring emergency intervention. Despite national-level regulations, including the ban on paraquat in Korea, pesticide ingestion continues to cause a substantial clinical burden [1]. In recent years, although deaths from highly toxic pesticides such as paraquat and methomyl have declined due to regulatory bans, there has been a relative increase in suicide attempts involving less irritating, water-soluble, and lower-toxicity herbicides. Notably, fatalities related to glyphosate- and glufosinate-containing herbicides have been steadily rising in Korea [2]. As a result, pesticide poisoning persists as a major clinical concern, often requiring intensive care and posing significant challenges in acute management [3].

Compared to other chemical exposures, pesticide poisoning is associated with considerably higher fatality rates, owing to its diverse toxicological mechanisms and frequent complications including acid–base imbalance, shock, arrhythmias, hypotension, and respiratory failure [3,4,5]. In addition, it is often difficult to accurately determine the type and amount of pesticide ingested, and the heterogeneity of clinical presentations makes early severity assessment and treatment decisions particularly challenging. Although few studies have provided detailed time-to-death distributions across pesticide types, existing evidence indicates that most acute fatalities occur within the first few days after ingestion, with many deaths occurring within 1–2 days depending on the compound [6]. Given the rapid progression and high lethality of many pesticide toxicities, early resuscitation and timely initiation of treatment are critical to improving clinical outcomes [7].

According to a nationwide study on poisoning trends based on Korea’s national emergency medical data, pesticides were identified as the leading cause of both emergency department and in-hospital mortality among poisoning cases. Given the continued presence of a sizable agricultural population, a significant reduction in pesticide-related deaths remains unlikely. In particular, pesticides are a major cause of death among elderly patients with poisoning, who often have poorer prognoses due to age-related physiological vulnerability. As such, timely evaluation and early therapeutic intervention are especially critical when pesticide poisoning is suspected in this population [8].

To support clinical decision making, several prognostic tools—such as APACHE II (Acute Physiologic and Chronic Health Evaluation II) score, PSS (Poisoning Severity Score), SAPS (Simplified Acute Physiology Score) and SOFA (Sequential Organ Failure Assessment)—have been used, along with key clinical indicators like mechanical ventilation and vasopressor use [5,9,10,11,12,13]. Although they are useful in general critical care populations, their predictive performance in pesticide-poisoned patients has been reported to be limited, with reported Area under the Receiving Operating Characteristic Curves (AUCs) ranging widely from 0.72 to 0.90. The Paraquat Poisoning Prognostic Score (PQPS) has been studied only in paraquat intoxication, while the PSS was designed for general toxic exposures and is not pesticide-specific. Likewise, APACHE II, SOFA, and SAPS II were originally developed for general ICU populations, and, although they have been applied to pesticide poisoning, they may not adequately reflect its distinct pathophysiology. Furthermore, as these tools were primarily developed for intensive care unit (ICU) patients based on data collected within first 24 h of ICU admission, they are not specifically tailored for early risk stratification in the emergency department setting, where rapid decision making is crucial [13,14,15]. Recent methodological studies in machine learning and time-series forecasting have also noted that increasing model complexity does not necessarily guarantee superior predictive performance, emphasizing the importance of balancing accuracy with interpretability in applied settings [16].

Given the continued clinical burden of pesticide poisoning and the challenge of early severity assessment, there is a need to improve prognostic tools that can help clinicians promptly identify high-risk patients. In this study, we aimed to develop and validate machine learning-based models to predict 14-day in-hospital mortality in patients with acute pesticide poisoning. Using only clinical and laboratory data available within the first 2 h of presentation, our goal is to enable prompt risk stratification and assist clinicians in early prognostication. Through this approach, we sought to support early prognostication and ultimately improve the care of pesticide-intoxicated patients.

## 2. Materials and Methods

### 2.1. Study Population and Study Design

This observational retrospective cohort study was conducted at Soonchunhyang University Cheonan Hospital from January 2015 to December 2020. Over the study period, a total of 1081 patients diagnosed with acute pesticide poisoning were admitted to the Institute of Pesticide Poisoning.

**Figure 1:**
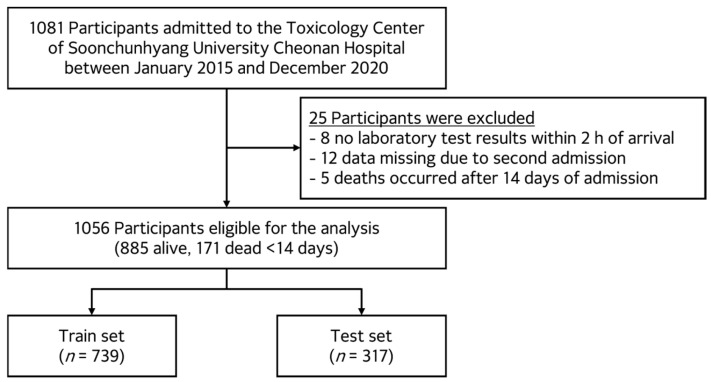
Flow diagram of participant inclusion and exclusion. Of the 1081 patients admitted to the Toxicology Center between January 2015 and December 2020, 25 were excluded due to missing laboratory results within 2 h of arrival (*n* = 8), missing data from secondary admissions (*n* = 12), or death occurring after 14 days of admission (*n* = 5). A total of 1056 patients were included in the final analysis (885 survivors, 171 non-survivors within 14 days). The dataset was randomly divided into train (*n* = 739) and test (*n* = 317) sets in a 7:3 ratio.

Patients were excluded if laboratory test results were unavailable within the first two hours of hospital arrival or if key data were missing due to repeated hospital visits. Additionally, patients who died more than 14 days after admission were excluded, as pesticide-related mortality typically occurs within this time frame, and later deaths were more likely attributable to non-toxicological causes. After applying these criteria, 1056 patients were included in the final analysis. Of these, 885 survived, and 171 died within 14 days of admission. A detailed flow diagram outlining the inclusion and exclusion criteria is presented in Figure 1.

**Figure 2:**
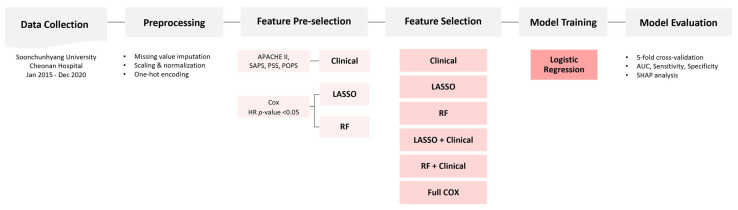
Schematic workflow of the prediction model development process for 14-day in-hospital mortality in acute pesticide poisoning. Following data collection and preprocessing, features were selected using both clinical knowledge and machine learning-based approaches (LASSO, random forest). Six feature sets were constructed and used to train logistic regression models. Model performance was evaluated via stratified 5-fold cross-validation using metrics including area under the curve (AUC), sensitivity, specificity, and SHAP (Shapley Additive exPlanations) analysis.

The study cohort was randomly divided into train and test sets in a 7:3 ratio. Stratified sampling was applied to maintain the distribution of pesticide types and mortality outcomes across both subsets. An overview of the feature selection, model development, and evaluation pipeline is illustrated in Figure 2.

This study was conducted in accordance with the ethical principles of the Declaration of Helsinki and was approved by the Institutional Review Board of Soonchunhyang University Cheonan Hospital (IRB number: 2020-02-016). The requirement for written informed consent was waived by the Institutional Review Board due to the retrospective nature of the study and the use of anonymized patient data.

### 2.2. Data Collection

Demographic and clinical data were extracted from electronic medical records (EMRs) and documented by attending physicians using standardized data collection forms. The timing of pesticide exposure and hospital arrival was determined through a review of emergency department charts. The ingested volume of pesticide was estimated based on the number of reported swallows, with each swallow approximated to be 20 mL, as previously described in toxicology studies. Information was primarily obtained from patient or caregiver recall, occasionally corroborated by emergency records or remaining containers. To minimize recall bias, ingestion volume was categorized into broad groups (<100 mL, 100–300 mL, >300 mL, unknown). Only laboratory test results obtained within the first two hours of admission were included in the analysis to ensure consistency in early clinical assessment.

### 2.3. Data Processing

All preprocessing procedures were conducted using Python (version 3.11) and relevant libraries, including pandas, scikit-learn, and NumPy. Among the collected variables, only those with a missing rate of less than 10% were included for model development. One exception was the variable “seizure,” which was retained despite exceeding this threshold, based on its known clinical relevance to acute outcomes.

Variables were classified as numerical or categorical based on their data type and the number of unique values. To accurately reflect each patient’s initial clinical condition, only the first available test values were used. Numerical variables underwent outlier removal using the 1st and 99th percentiles and were subsequently rescaled to maintain unit consistency. Variables with skewed distributions—including procalcitonin (PCT), ethanol, creatine kinase (CK), CK-MB, and C-reactive protein (CRP)—were log-transformed.

Additional variables were rescaled based on clinical interpretability: specific gravity (SG) was multiplied by 1000; pH and PT-INR by 10; and triglycerides (TG), glucose, partial pressure of oxygen (pO_2_), partial pressure of carbon dioxide (pCO_2_), and platelet count were divided by 10. These steps were applied only to adjust the numeric range for easier interpretation of regression coefficients, without affecting model performance. All continuous variables were subsequently standardized using the StandardScaler, so prior rescaling only changed the units of coefficients without altering data distribution or model discrimination.

Categorical variables—including comorbidities, neurological symptoms, and urinalysis results—were binarized based on clinical relevance. Urine dipstick findings were semi-quantitatively encoded, while urinary red and white blood cell counts were categorized into ordinal tiers. Sex and smoking status were standardized and subsequently binarized or one-hot encoded. Pesticide type and estimated dose were also converted to dummy variables via one-hot encoding, with glufosinate and the lowest dose category designated as reference groups to prevent multicollinearity. Glufosinate was chosen due to its sufficient sample size and a median-level mortality rate among pesticides.

Troponin-T values were dichotomized using the clinical cut-off of 0.014 ng/mL, which corresponds to the standardized 99th percentile threshold for myocardial injury. Once this threshold is exceeded, the absolute magnitude of elevation reflects diverse conditions and is less interpretable as a continuous measure. Therefore, the variable was modeled as binary to reflect its established clinical significance [17].

The final feature set included scaled continuous variables, log-transformed biomarkers, binary indicators, categorized urine test values, and one-hot encoded categorical features. Redundant or original unprocessed variables were excluded from model input.

For machine learning implementation, Boolean variables were converted to integers (0 or 1). Missing values in categorical variables were imputed using the most frequent category. For numerical variables, the Multivariate Imputation by Chained Equations (MICE) method was applied. Finally, all continuous variables were standardized using the StandardScaler function from scikit-learn.

### 2.4. Variable Selection

Candidate variables were initially screened using univariable Cox proportional hazards regression (*p* < 0.05), which evaluates the association between each variable and mortality based on time-to-event analysis. Following this initial filtering step, three distinct feature selection strategies were applied to construct the final feature sets: (1) clinical relevance, (2) Least Absolute Shrinkage and Selection Operator (LASSO) regularization, and (3) Random Forest (RF) importance ranking.

The clinically curated list was developed based on variables incorporated in established prognostic scoring systems commonly used in critically ill or poisoned patients, including APACHE II, SAPS, PSS, and POPS. Frequently used components of these scores—such as vital signs, neurological status, and laboratory values—were selected to ensure a meaningful and interpretable comparator [9,11,18]. Certain variables, including serum sodium and hematocrit, were retained in this list despite not reaching statistical significance in the univariable Cox analysis, due to their recognized roles in clinical assessment tools. In addition, chronic comorbidities such as diabetes mellitus (DM), chronic kidney disease (CKD), and hypertension (HTN) were also included to reflect baseline patient risk.

The LASSO-based feature set was constructed by applying L1-regularized logistic regression (LR) using the scikit-learn implementation of the LASSO algorithm. This method imposes a penalty on the absolute magnitude of regression coefficients, which effectively reduces less informative variables to zero, thereby yielding a sparse and interpretable model.

The RF-based feature set was derived by training a Random Forest classifier on the training data and selecting features with a mean decrease in impurity (MDI) of ≥0.01. This threshold identified variables with consistent and meaningful importance across decision trees in the ensemble. Features that demonstrated robust importance across bootstrapped samples were retained in the final list.

All preprocessing procedures and feature selection strategies were applied exclusively to the training dataset to prevent data leakage and to ensure the generalizability of model evaluation on the test set.

### 2.5. Prediction Model Construction and Selection

We developed prediction models using LR. The moderate sample size and lack of external validation required a model with low overfitting risk and high generalizability. Compared to more complex machine learning algorithms, LR offers greater transparency and robustness, making it particularly suitable for small, heterogeneous clinical datasets—such as those involving diverse pesticide types and variable exposure profiles. The mechanistic heterogeneity across different pesticide classes poses challenges in modeling due to increased data sparsity and dimensionality when incorporating pesticide-specific variables. In such scenarios, linear models like LR are generally more resilient to overfitting and better equipped to handle sparse, high-dimensional data. Moreover, LR provides interpretable model coefficients and odds ratios (ORs), which facilitate clinical applicability and support decision making at the bedside. Based on these advantages, LR was selected as the primary modeling approach [19,20,21].

Six distinct feature sets were constructed based on Cox-filtered variables and three selection strategies: (1) full Cox-filtered feature set, (2) clinically curated features based on established prognostic scoring systems, (3) features selected via LASSO regularization, (4) a combination of LASSO selected and clinical features, (5) features ranked by R importance, and (6) a combination of RF-ranked and clinical features.

For each feature set, stratified 5-fold cross-validation was employed to preserve the distribution of the outcome variable and pesticide types across folds. Hyperparameter tuning was integrated within the cross-validation loop to determine the optimal model configuration. The best-performing model for each feature set was retrained using the entire training dataset and finalized with its corresponding optimal probability threshold.

Model performance was assessed on an independent test dataset. Data imputation and scaling procedures were applied using transformation parameters derived solely from the training set to prevent data leakage. To evaluate model robustness and generalizability, 1000 bootstrap resamples of the test set were performed, and 95% confidence intervals (CIs) for the area under the receiver operating characteristic curve (AUC) were computed. Final model evaluation considered test set AUC, number of features used, predictive accuracy, model simplicity, and performance stability, providing a comprehensive basis for model selection.

### 2.6. Study Outcome

The primary outcome variable for model prediction was in-hospital mortality within 14 days of admission. This 14-day window was selected based on the clinical characteristics of pesticide poisoning and the temporal distribution of deaths observed in this cohort. Mortality events occurring beyond 14 days were rare and were considered more likely to result from non-toxicological complications. Therefore, to minimize confounding from unrelated causes, patients who died after the 14-day period were excluded from the analysis. Patients who were discharged or lost to follow-up within 14 days and had no documented evidence of death were assumed to have survived and were included in the survival group. In total, 1056 patients were analyzed, including 171 in-hospital deaths (16.2%) within 14 days and 885 survivors.

### 2.7. Statistical Analysis

All statistical analyses were conducted using Python (version 3.11.13; Python Software Foundation, https://www.python.org/) with the following libraries: scikit-learn for machine learning, statsmodels for statistical inference, lifelines for survival analysis, and shap for model interpretability. Model performance was evaluated using standard classification metrics, including area under the receiver operating characteristic curve (AUC), sensitivity, specificity, precision, and F1 score. Pairwise comparisons of AUC values between models were conducted using DeLong’s test to assess statistical differences in predictive performance. To interpret the contribution of individual features to model predictions, SHapley Additive exPlanations (SHAP) values were computed. A two-sided *p*-value of <0.05 was considered statistically significant for all hypothesis testing.

## 3. Results

### 3.1. Study Population and Baseline Characteristics

Baseline demographic and clinical characteristics of the study population are summarized in Table 1. The mean age of patients was 63.0 ± 15.3 years, and 63.1% were male. The estimated volume of ingested pesticide was relatively evenly distributed across the following categories: 50–100 mL (19.2%), 100–200 mL (17.8%), 200–300 mL (15.5%), and >300 mL (11.6%). The overall 14-day in-hospital mortality rate was 16.2% (171 of 1056), with no statistically significant difference between the training and test sets (*p* = 1.000). Among the pesticide types, glufosinate (21.6%) and glyphosate (22.2%) were the most ingested agents, followed by paraquat (12.6%) and organophosphates (10.5%). No significant differences were observed in baseline clinical variables—such as vital signs and laboratory parameters—between the training and test datasets. The mean initial Glasgow Coma Scale (GCS) score was 12.7 ± 3.6, and the average initial systolic blood pressure was 132.2 ± 27.1 mmHg. Further details on baseline comorbidities, laboratory test results, and additional clinical characteristics are provided in Supplementary Table S1.

**Figure 3:**
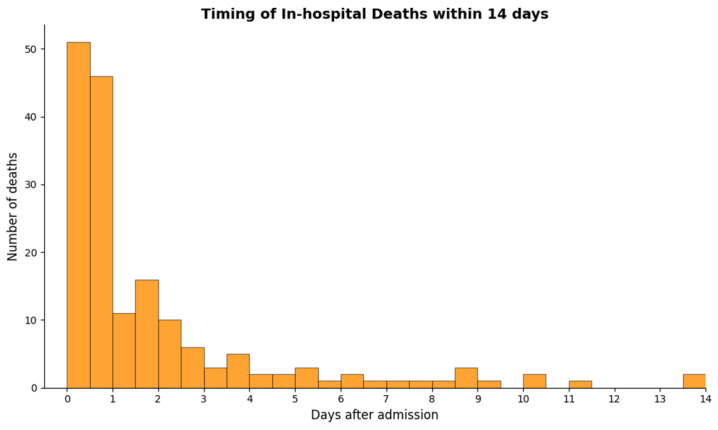
Distribution of in-hospital deaths by time from admission among patients with acute pesticide poisoning. The histogram, plotted using 0.5-day (12-h) bins, shows the timing of 171 deaths that occurred within 14 days of hospital admission out of a total cohort of 1056 patients. A prominent early peak is observed within the first 24 h (97 deaths), followed by a gradual decline, with most deaths concentrated in the first three days.

Figure 3 illustrates the temporal distribution of in-hospital deaths following admission via the emergency department. Among a total of 1056 patients, 171 (16.2%) died within 14 days. A marked peak in mortality was observed within the first 24 h (97, 56.7%), followed by a gradual decline over the subsequent days, with most deaths occurring within the initial three days (140, 81.9%). No additional distinct mortality peaks were noted during the 14-day observation window.

**Figure 4:**
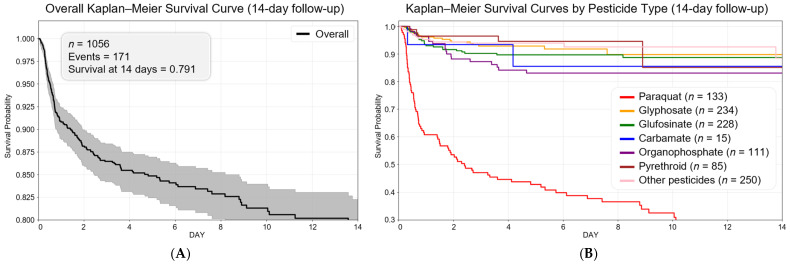
Kaplan–Meier survival curves for 14-day all-cause in-hospital mortality among patients with acute pesticide poisoning. (**A**) Overall survival curve (*n* = 1056): the estimated 14-day survival probability was 0.791, with most deaths occurring within the first 3 days. (**B**) Pesticide-specific survival curves: paraquat exposure showed the steepest decline in survival, with a median survival time of 2.4 days. No median survival was reached for other pesticide types within the 14-day observation period.

Figure 4 displays the Kaplan–Meier survival curves for the study cohort. The overall 14-day survival probability was 0.791. The mean observation duration was 6.0 days, and the median observation period was 4.5 days. The median survival time was not reached for the overall cohort. However, among pesticide types, paraquat was the only agent with a defined median survival of 2.4 days, indicating substantially higher early mortality compared to other pesticides, for which the 14-day median survival was not reached.

### 3.2. Selected Variables

Variables for model development were selected using four distinct approaches: univariable Cox proportional hazards regression (*p* < 0.05), LASSO regression, random forest (RF) feature importance ranking, and clinical relevance based on established prognostic indicators.

The variables identified by each method are summarized in Supplementary Table S2. Based on these selection strategies, six distinct feature sets were constructed for model training and comparative evaluation: (1) Full Cox (*n* = 53), (2) LASSO (*n* = 22), (3) RF (*n* = 36), (4) LASSO + Clinical (*n* = 40), (5) RF + Clinical (*n* = 48), and (6) Clinical (*n* = 25).

### 3.3. Model Performance and Interpretation

#### 3.3.1. Model Performance

As summarized in Table 2, we evaluated the predictive performance of LR models developed using each of the six predefined feature sets. Performance was compared based on area under the receiver operating characteristic curve (AUC) and the number of features included in each model.

Among all configurations, the LASSO + Clinical feature set (Set 4) demonstrated the highest overall performance, achieving an AUC of 0.926, balanced accuracy of 0.843, sensitivity of 0.824, specificity of 0.861, and an F1 score of 0.646. Pairwise comparisons of AUCs were conducted using DeLong’s test. The difference in AUC between the LASSO + Clinical model (Set 4) and the LASSO-only model (Set 2) was not statistically significant (*p* = 0.55), whereas all other comparisons yielded statistically significant differences.

Considering its comparable performance and reduced number of features, the LASSO-only model (Set 2) was selected as the final model. This model achieved an AUC of 0.923, balanced accuracy of 0.835, sensitivity of 0.804, specificity of 0.865, and an F1 score of 0.641.

The optimal classification threshold was determined to be 0.450, selected based on the maximum F1.5 score—a weighted harmonic mean of precision and recall that places greater emphasis on recall (β = 1.5). As shown in Supplementary Figure S1, at this threshold the LASSO model yielded an F1.5 score of 0.714, sensitivity of 0.843, specificity of 0.857, and maintained an AUC of 0.923. This threshold was chosen to minimize false negatives and thereby reduce the risk of missed opportunities for timely intervention in a population with a relatively low event rate.

Model performance stratified by pesticide type is presented in Table 3. The mean AUC across pesticide categories was 0.896, with the highest performance observed for pyrethroids (AUC = 1.000) and the lowest for glyphosate (AUC = 0.803). However, the estimate for the pyrethroid group is based on a small subgroup (*n* = 26, 2 deaths) and thus has limited statistical reliability. Statistically meaningful predictive performance was achieved across all pesticide groups, except for carbamates, which were excluded due to a small sample size (*n* < 15). Detailed performance metrics by pesticide type are shown in Supplementary Figure S2.

#### 3.3.2. Feature Importance Analysis via SHAP and Model Coefficients

**Figure 5:**
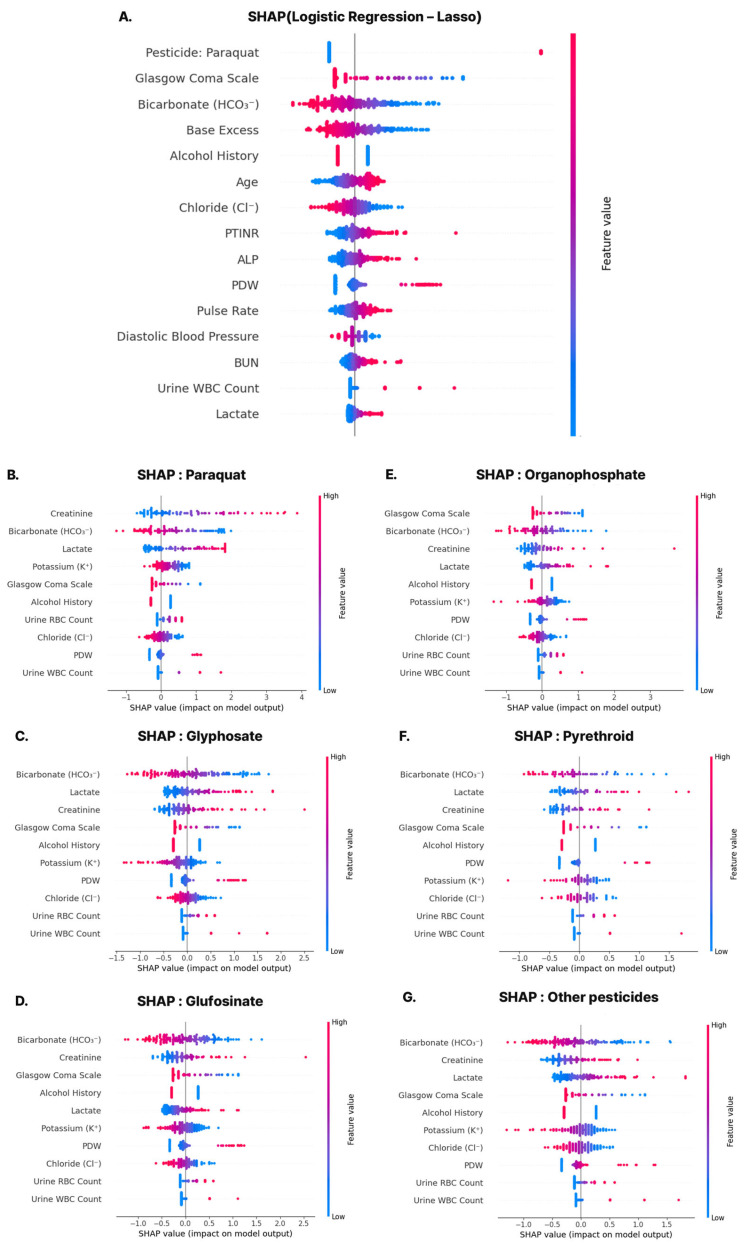
SHAP summary plots identifying top predictors of 14-day mortality in the logistic regression models. (**A**) Logistic regression model with LASSO, (**C**–**G**) Stratified SHAP analysis by pesticide type: Paraquat (**B**), Glyphosate (**C**), Glufosinate (**D**), Organophosphate (**E**), Pyrethroid (**F**), Other pesticides (**G**). Features are ranked by their mean absolute SHAP values, reflecting their overall importance in the model. Color indicates feature values (red: high, blue: low). Neurological status (Glasgow Coma Scale), acid–base markers (bicarbonate, base excess, lactate), and paraquat ingestion consistently emerged as top predictors across subgroups.

To interpret the contribution of individual variables to 14-day mortality prediction, both SHapley Additive exPlanations (SHAP) values and LR coefficients from the final LASSO model were analyzed. As shown in Figure 5, the SHAP summary plot of the base LASSO model identified paraquat ingestion, lower Glasgow Coma Scale (GCS) scores, and decreased bicarbonate (HCO_3_^−^) levels as the most influential predictors associated with increased mortality risk. Notably, the absence of a history of alcohol use was also linked to higher predicted risk.

In the pesticide-specific SHAP analysis (excluding carbamates due to a limited sample size, *n* < 15), paraquat ingestion, GCS, HCO_3_^−^, base excess (BE), and alcohol history consistently emerged as key predictors of 14-day mortality. Among non-paraquat pesticide exposures, GCS was the most influential variable, underscoring the prognostic value of initial neurological status. Other frequently ranked features included age, platelet distribution width (PDW), serum chloride (Cl), and prothrombin time-international normalized ratio (PT-INR), highlighting their relevance across various pesticide subtypes.

To isolate the contribution of clinical features independent of pesticide class, SHAP values were recalculated after removing the pesticide type variable from the model. Results are presented in Supplementary Table S3. In paraquat poisoning, creatinine, HCO_3_^−^, and lactate were the most influential variables. For glyphosate and glufosinate exposures, HCO_3_^−^ consistently ranked highest, followed by GCS, creatinine, and lactate. In pyrethroid poisoning, HCO_3_^−^, lactate, and GCS were again top contributors. Organophosphate cases showed the highest SHAP values for GCS, followed by HCO_3_^−^ and creatinine. These patterns collectively underscore the universal importance of acid–base balance and neurological status in predicting mortality across different pesticide types.

Additionally, several features—including alcohol history, serum potassium, PDW, Cl, and urinary abnormalities such as erythrocyte and leukocyte counts—demonstrated consistent contributions across multiple pesticide groups, indicating their potential utility for clinical risk stratification.

The SHAP summary of the LASSO + Clinical model revealed broadly similar patterns. However, several additional features—such as hypertension (HTN), pH, mean arterial pressure (MAP), anion gap, alanine aminotransferase (ALT), total bilirubin, and underlying pulmonary disease—were ranked within the top 15 in terms of SHAP values (Supplementary Figure S3). In contrast, features such as age, Cl, PDW, pulse rate (PR), blood urea nitrogen (BUN), urine leukocyte count, and lactate, which were highly ranked in the base LASSO model, had reduced importance in the combined model.

The final LR model yields a linear predictor composed of 22 features selected via LASSO, as follows:

Z = −1.643960 + 3.677636 × pest_paraquat − 0.664450 × GCS_total_est − 0.530746 × HCO_3_^−^ − 0.520141 × alcohol_bin − 0.445294 × BE + 0.377111 × PDW + 0.290795 × PTINR_scaled − 0.276784 × Cl + 0.273494 × age + 0.262155 × Uleuko_num + 0.242221 × ALP + 0.198176 × PR_adm − 0.166996 × DBP_adm + 0.153654 × BUN + 0.131072 × lactate_ER + 0.118909 × RDW − 0.116229 × O2sat − 0.100602 × Kal − 0.090679 × MCHC + 0.089919 × Cr + 0.080628 × Uery_num − 0.034055 × Gluc_scaled (1)

In this model, paraquat ingestion had the largest positive coefficient (β = 3.678; OR = 39.6), indicating a markedly elevated risk of mortality, as shown in Supplementary Table S4. Other variables with positive coefficients and odds ratios (OR) > 1 included elevated PT-INR, older age, higher BUN, and elevated lactate levels—all significantly associated with increased mortality risk.

In contrast, higher GCS scores, higher bicarbonate levels, and a history of alcohol use were significantly associated with lower mortality risk and acted as protective factors. Likewise, elevated BE, higher Cl levels, and higher diastolic blood pressure (DBP) also showed negative regression coefficients and OR below 1, indicating protective associations with reduced mortality. These findings were consistent with the SHAP-based importance rankings, reinforcing the clinical interpretability and reliability of the model.

#### 3.3.3. Model Calibration and Risk Stratification

**Figure 6:**
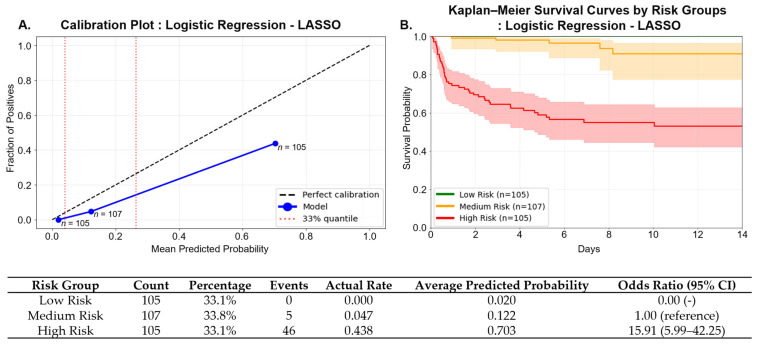
Calibration and survival analysis of the logistic regression model with LASSO-selected features. (**A**) Calibration plot of the LASSO-based logistic regression model. Patients were stratified into low-, medium-, and high-risk groups based on tertiles of predicted probability (cutoffs at 0.039 and 0.264). Blue dots indicate observed event rates for each group; the dashed line represents perfect calibration. The Brier score was 0.1051, indicating good overall calibration. (**B**) Kaplan–Meier survival curves for 14-day in-hospital mortality stratified by risk group. Shaded regions represent 95% confidence intervals. Event rates were 0.0% (*n* = 105), 4.7% (*n* = 107), and 43.8% (*n* = 105) for the low-, medium-, and high-risk groups, respectively. Median survival was not reached in any group. The log-rank test revealed significant differences between high- vs. medium-risk and high- vs. low-risk groups (both *p* < 0.001); the difference between medium- and low-risk groups was not statistically significant (*p* = 0.089).

As shown in Figure 6, patients were stratified into low-, medium-, and high-risk groups based on tertiles of predicted mortality probability, with cut-off values at 0.039 and 0.264 corresponding to the 33rd and 67th percentiles. The observed mortality rates were 0.0%, 4.7%, and 43.8% for the low-, medium-, and high-risk groups, respectively, while the mean predicted probabilities were 0.020, 0.122, and 0.703. These findings suggest that the model systematically overestimated absolute risk across all strata.

Using the medium-risk group as the reference, the odds ratios (ORs) for the low- and high-risk groups were 0.00 (95% CI: not estimable) and 15.91 (95% CI: 5.99–42.25), respectively. To quantitatively assess calibration performance, we calculated the Brier score, which measures the mean squared difference between predicted probabilities and actual outcomes. The model achieved a Brier score of 0.1051, indicating excellent calibration. This low value demonstrates strong agreement between predicted probabilities and observed mortality rates, complementing the visual calibration assessment shown in Figure 6A.

Kaplan–Meier survival curves stratified by risk group demonstrated clear separation across the groups (Figure 6B). Although the median survival time was not reached in any group during the 14-day observation period, statistically significant differences in survival were observed between the high- vs. medium-risk and high- vs. low-risk groups (both log-rank *p* < 0.001). The difference between the medium- and low-risk groups did not reach statistical significance (log-rank *p* = 0.089).

Baseline characteristics across the three risk groups are summarized in Supplementary Table S5. Compared with the low-risk group, patients in the high-risk group were substantially older (mean age: 70.0 vs. 53.9 years), exhibited lower GCS scores (10.5 vs. 14.7), higher APACHE II scores (16.0 vs. 5.3), and lower systolic and diastolic blood pressures (SBP: 125.7 vs. 138.5 mmHg; DBP: 72.1 vs. 81.7 mmHg).

In addition, key laboratory values were significantly elevated in the high-risk group: BUN (18.0 vs. 13.3 mg/dL), serum creatinine (1.3 vs. 0.8 mg/dL), anion gap (21.8 vs. 14.0 mEq/L), and serum lactate (5.60 vs. 2.00 mmol/L). Notably, the prevalence of paraquat poisoning was markedly higher in the high-risk group (37.1%) compared to the low-risk group (0.0%), which is consistent with its known lethality. Furthermore, the proportion of patients with a history of alcohol use was lower in the high-risk group (37.1% vs. 61.0%), consistent with prior findings from the SHAP analysis.

### 3.4. Comparison with APACHE II Scoring Model

To benchmark the performance of our machine learning model, we evaluated the predictive capability of the APACHE II scoring system using the same dataset. The APACHE II score—calculated from clinical and laboratory data obtained within the first 24 h of admission—was used as the sole predictor in a univariable LR model.

This model achieved a test AUC of 0.835 (95% CI: 0.781–0.888), with a balanced accuracy of 0.746, precision of 0.387, and an F1 score of 0.500 at a decision threshold of 0.150. This threshold was chosen to maximize recall, consistent with the strategy applied to our final model (see Supplementary Table S6). The distribution of APACHE II scores within the study cohort is illustrated in Supplementary Figure S4.

## 4. Discussion

Most variables included in the predictive model developed in this study align with established clinical prognostic indicators. Notably, variables such as Glasgow Coma Scale (GCS), bicarbonate (HCO_3_^−^), base excess (BE), pulse rate (PR), diastolic blood pressure (DBP), oxygen saturation (O_2_sat), blood urea nitrogen (BUN), serum creatinine (Cr), lactate, red cell distribution width (RDW), potassium (Kal), and chloride (Cl) are commonly incorporated into critical care scoring systems such as APACHE II and SAPS. These features also overlapped with the predefined clinically curated list, suggesting that both clinical interpretability and statistical significance were considered during model development.

A particularly interesting finding was the inclusion of the variable ‘alcohol_bin’, which reflects the patient’s history of alcohol use. In this model, a negative regression coefficient indicated a protective association with 14-day mortality. This finding contrasts with conventional clinical expectations, which associate chronic alcohol use with worse outcomes due to immune suppression, hepatic dysfunction, and nutritional deficits. However, previous studies have primarily focused on acute co-ingestion of alcohol with pesticides, rather than evaluating the effects of chronic alcohol use as an independent risk factor [22]. In this study, chronic alcohol use was defined only as a binary alcohol history, without accounting for amount, duration, or drinking pattern, which may have introduced measurement errors. The counterintuitive protective association may also reflect unmeasured confounding (e.g., age, socioeconomic status, medical access, or poisoning intent) or biases such as survivorship or self-reporting, rather than a true biological effect. Future research should refine the characterization of alcohol use and reassess its predictive role through external validation.

The positive regression coefficients for urinary leukocytes (‘Uleuko_num’) and erythrocytes (‘Uery_num’) suggest that elevations in these parameters on initial urinalysis may serve as early markers of acute kidney injury (AKI) or systemic inflammatory response in patients with pesticide poisoning. Perazella (2015) emphasized the diagnostic and prognostic value of urinary findings such as tubular cells, RBCs, and WBCs in AKI [23], and studies such as the Yale PhD thesis have demonstrated that sediment-based scoring predicts mortality in AKI populations [24]. Although pesticide-specific evidence remains limited, these findings support the use of early urinary abnormalities as valuable predictors of renal stress and inflammation in poisoning contexts, potentially enhancing the predictive accuracy of our model. However, these associations may also reflect confounding factors such as asymptomatic pyuria, pre-existing urinary tract infection, or chronic kidney disease, and thus require cautious interpretation.

Several variables that showed significant differences across predicted risk groups—such as age, paraquat exposure, GCS, HCO_3_^−^, creatinine, BUN, PT-INR, chloride, potassium, and lactate—were also retained in the final model. This concordance reinforces the clinical validity of the selected features and suggests that the model effectively captures key prognostic signals. Among these, paraquat, a highly lethal herbicide banned in many countries, exhibited one of the highest regression coefficients and SHAP values. While this raises concerns regarding paraquat-driven bias, subgroup analyses showed robust model performance across other pesticide types, mitigating concerns about over-reliance on a single agent. Additionally, some statistically significant variables—such as body temperature, AST, and anion gap—were excluded, likely due to multicollinearity or lower independent predictive value, highlighting the importance of data-driven feature selection.

Compared to our model, the APACHE II–based model achieved a test AUC of 0.835 with moderate overall balanced accuracy (0.746) and sensitivity (0.706). However, its precision was limited (0.387), resulting in a relatively low F1 score (0.500). These findings suggest that, while APACHE II can detect a substantial proportion of mortality events, its lower precision limits its utility in triaging pesticide-poisoned patients. This limitation likely stems from the non-specific nature of APACHE II, which was developed for general ICU populations and may not account for the distinct pathophysiology of pesticide poisoning. In contrast, our model, based on LASSO- and clinically selected features, demonstrated superior discriminatory power and interpretability, making it more suitable for mortality risk stratification in this specialized population. Our study compared the model mainly with APACHE II, a widely used general prognostic tool, but a broader comparison with recently developed machine learning-based models in toxicology would provide additional context. Direct comparisons were not feasible in this study because most existing models focus on different toxic agents or rely on variables and outcomes that are not directly comparable to acute pesticide poisoning. Future work should therefore include head-to-head evaluations with these emerging models to more clearly delineate the strengths and limitations of our approach.

From a clinical perspective, our model could be integrated into emergency department workflows as a decision-support tool. Using only clinical and laboratory data from the first 2 h, the model could be embedded within electronic medical records to generate risk scores. In our cohort, tertile-based stratification identified a high-risk group (≥0.265) characterized by substantially increased short-term mortality, who might warrant early ICU consultation, closer monitoring, or timely interventions such as hemoperfusion, while low-risk patients could receive standard care.

Recent time-series methods, including the TCN-Linear model, have reported good performance in chaotic or financial forecasting [16]. These findings suggest that increasing model complexity does not always lead to better predictive accuracy. However, such approaches have not been tested in clinical risk prediction and do not address essential requirements such as interpretability, calibration, or external validation. For this reason, we chose logistic regression as a transparent first step. Temporal models may be explored in future studies, but only if they can remain interpretable and clinically reliable.

This study has several limitations. First, it was a retrospective single-center study, which may limit the generalizability of the findings and does not exclude potential biases inherent to retrospective designs. Although national reimbursement policies and clinical guidelines in Korea may reduce inter-institutional variability, external validation with independent datasets—ideally through multi-center prospective cohorts with real-time data collection—is needed to confirm the robustness and reproducibility of our model in clinical practice. Second, although we selected 14-day in-hospital mortality as the primary outcome based on our cohort’s death distribution, standardized outcome windows for pesticide poisoning have not been established. Therefore, caution is needed when generalizing our 14-day endpoint. Third, while LR was chosen for its interpretability and low risk of overfitting, alternative machine learning models—such as random forest, gradient boosting, or deep learning—may offer improved predictive performance. Future studies should explore these approaches, but, given the heterogeneous mechanisms of different pesticides and the small sample size for some agents, simpler models such as logistic regression may be preferable to avoid overfitting. Ideally, these methods should be combined with explainability techniques such as SHAP or LIME to balance accuracy with interpretability. Lastly, the relatively low precision reflects dataset imbalance and the trade-off between sensitivity and specificity. In clinical settings, we prioritized sensitivity to minimize the risk of missing high-risk patients, even at the expense of precision. This limitation should be acknowledged, and future studies should consider advanced resampling or class-weighting strategies.

## 5. Conclusions

In this retrospective cohort study involving over 1000 patients with pesticide poisoning, we developed and validated an interpretable machine learning model to predict in-hospital mortality within 14 days of admission. By combining features selected through LASSO regularization and clinical relevance, our LR-based model demonstrated superior performance (AUC 0.923) compared to conventional tools such as the APACHE II score. The final model achieved high predictive accuracy, preserved interpretability through odds ratio estimation, and maintained generalizability across diverse pesticide types. Key prognostic indicators—including GCS, bicarbonate, lactate, creatinine, and base excess—were consistently identified as top predictors, reinforcing their pathophysiological relevance in acute toxicological injury. Importantly, the model’s robust performance across pesticide subtypes and its clinically grounded feature set suggests strong utility in real-world settings, particularly for timely triage and resource allocation. While paraquat exposure contributed significantly to overall mortality, subgroup analyses confirmed that the model retained predictive validity even in non-paraquat and unknown-agent cases. By integrating clinical interpretability, computational rigor, and pathophysiological insight, our approach provides a valuable tool for improving acute care in toxicology. Future multicenter and prospective studies are warranted to externally validate and further refine this model, with the goal of embedding it into clinical decision support systems for emergency and critical care settings.

## Figures and Tables

**Table 1:** Baseline demographic and clinical characteristics of study participants.

Variable	All Patients (*n* = 1056)	Train Set (*n* = 739)	Test Set (*n* = 317)	*p*-Value
Age, years	63.0 ± 15.3	62.9 ± 15.3	63.3 ± 15.3	0.74
Sex, male (%)	666 (63.1)	462 (62.5)	204 (64.4)	0.62
Body mass index, kg/m^2^	22.7 ± 3.1	22.7 ± 3.1	22.7 ± 3.1	0.99
Clinical history				
Diabetes (%)	194 (18.4)	131 (17.7)	63 (19.9)	0.46
Hypertension (%)	394 (37.3)	267 (36.1)	127 (40.1)	0.25
Lung disease (%)	92 (8.7)	67 (9.1)	25 (7.9)	0.61
Cardiac disease (%)	66 (6.2)	46 (6.2)	20 (6.3)	1.00
Chronic kidney disease (%)	13 (1.2)	8 (1.1)	5 (1.6)	0.72
Neuropsychiatric disease (%)	201 (19.0)	140 (18.9)	61 (19.2)	0.98
Pesticide category				
Glufosinate	228 (21.6)	160 (21.7)	68 (21.5)	1.00
Glyphosate	234 (22.2)	164 (22.2)	70 (22.1)	1.00
Organophosphate	111 (10.5)	78 (10.6)	33 (10.4)	1.00
Carbamate	15 (1.4)	10 (1.4)	5 (1.6)	1.00
Pyrethroid	85 (8.0)	59 (8.0)	26 (8.2)	1.00
Paraquat	133 (12.6)	93 (12.6)	40 (12.6)	1.00
Other pesticides	250 (23.7)	175 (23.7)	75 (23.7)	1.00
Amount of ingestion				
50–100 mL	203 (19.2)	149 (20.2)	54 (17.0)	1.00
100–200 mL	188 (17.8)	134 (18.1)	54 (17.0)	1.00
200–300 mL	164 (15.5)	108 (14.6)	56 (17.7)	1.00
>300 mL	150 (14.2)	101 (13.7)	49 (15.5)	1.00
Unknown	135 (12.8)	102 (13.8)	33 (10.4)	1.00
Death in 14 days	171 (16.2)	120 (16.2)	51 (16.1)	1.00
Social history				
Alcohol	483 (45.7)	343 (46.4)	140 (44.2)	0.55
Smoking, current	338 (32.0)	248 (33.6)	90 (28.4)	0.12
Smoking, ex-smoker	43 (4.1)	30 (4.1)	13 (4.1)	1.00
Vital Sign				
Systolic BP, mmHg	132.2 ± 27.1	132.1 ± 27.0	132.5 ± 27.3	0.82
Diastolic BP, mmHg	77.0 ± 14.4	77.0 ± 14.4	77.0 ± 14.4	0.99
PR,/min	87.8 ± 16.1	87.5 ± 16.5	88.5 ± 15.2	0.37
RR, breaths/min	19.0 ± 3.8	18.9 ± 3.9	19.0 ± 3.7	0.81
Body temperature, °C	36.3 ± 0.8	36.3 ± 0.8	36.3 ± 0.8	0.86
Glasgow Coma Scale	12.7 ± 3.6	12.7 ± 3.6	12.7 ± 3.6	0.96
BUN, mg/dL	16.1 ± 6.9	16.2 ± 7.2	15.7 ± 6.2	0.27
Creatinine, mg/dL	1.0 ± 0.6	1.0 ± 0.6	1.0 ± 0.5	0.54
HCO_3_^−^, mmol/L	21.1 ± 5.0	20.9 ± 5.0	21.4 ± 5.0	0.19
Anion Gap	17.8 ± 6.0	18.0 ± 6.1	17.4 ± 5.9	0.11
Lactate, mmol/L	2.70 (1.50, 4.80)	2.70 (1.50, 4.80)	2.80 (1.60, 4.70)	0.54

Values are expressed as mean ± standard deviation, median (interquartile range), or frequency (percentage), as appropriate. *p*-values reflect comparisons between the training and test datasets. “Other pesticides” include acetanilide, acetylaniline, alryoxylcarboxide, amide, anilin, arsenic, (aryloxy)phenopropionate, benzohydrazide, benzoate, chlorfenapyr, chloroacetamide, chloronicotinyl, diamide, diazine, dinitroaniline, endosulfan, fungicide, insect growth regulator, lambda-cyhalothrin, neonicotinoid, niacin, oxadiazole, phenoxy, pyrol, sulfonylurea, sulfoximine, sulfuryl fluoride, tetramic acid, tetrazolium oxide, urea, and unknown pesticides. Abbreviations: BP, blood pressure; PR, pulse rate; RR, respiratory rate; BUN, blood urea nitrogen; HCO_3_^−^, bicarbonate.

**Table 2:** Performance comparison of logistic regression models using different feature selection strategies on the test set.

Feature Set (*n*)	AUC (95% CI)	Balanced Accuracy	Sensitivity	Specificity	Precision	F1	*p*-Value with LASSO Model AUC
LASSO + Clinical (40)	0.926 (0.890–0.957)	0.843	0.824	0.861	0.532	0.646	0.55
LASSO (22)	0.923 (0.884–0.955)	0.835	0.804	0.865	0.533	0.641	ref
RF + Clinical (48)	0.9082 (0.862–0.947)	0.825	0.784	0.865	0.526	0.630	0.00 *
Full Cox (53)	0.9019 (0.856–0.941)	0.837	0.824	0.850	0.512	0.632	0.00 *
RF (36)	0.9019 (0.853–0.943)	0.807	0.745	0.868	0.521	0.613	0.00 *
Clinical (25)	0.8786 (0.823–0.924)	0.772	0.686	0.857	0.480	0.480	0.00 *

Model performance was evaluated using area under the receiver operating characteristic curve (AUC), balanced accuracy, sensitivity, specificity, precision, and F1 score. AUC values marked with an asterisk (*) are significantly different from the LASSO-based model (feature set 2) according to DeLong’s test (*p* < 0.05). Abbreviations: AUC, area under the receiver operating characteristic curve; LASSO, least absolute shrinkage and selection operator; Full Cox, model including all features with *p* < 0.05 in univariable Cox regression.

**Table 3:** Performance of the final logistic regression model using LASSO-selected features for each pesticide type.

Pesticide	*n*	Events (%)	AUC	Accuracy	Sensitivity	Specificity	Precision	F1 Score
Pyrethroid	26	2 (7.7)	1.000	1.000	1.000	1.000	1.000	1.000
Glyphosate	70	5 (7.1)	0.803	0.871	0.400	0.908	0.250	0.308
Glufosinate	68	7 (10.3)	0.864	0.838	0.714	0.852	0.357	0.476
Paraquat	40	26 (65.0)	0.901	0.725	1.000	0.214	0.703	0.825
Organophosphate	33	5 (15.2)	0.900	0.879	0.800	0.893	0.571	0.667
Other pesticides	75	5 (6.7)	0.906	0.867	0.600	0.886	0.273	0.375

Abbreviations: LASSO, least absolute shrinkage and selection operator; AUC, area under the receiver operating characteristic curve.

## Data Availability

The datasets used and/or analyzed during the current study are available from the corresponding author upon reasonable request.

## Supplementary Materials

The following supporting information can be downloaded at: https://www.mdpi.com/article/10.3390/toxics13100893/s1, Table S1: Baseline Characteristics; Table S2: Feature List; Full Cox, Clinical, LASSO, RF List; Figure S1: Model Performance Metrics Across Thresholds; Figure S2: Performance Metrics of the Pesticide-specific 14-day Mortality Prediction Models at a Threshold of 0.45; Table S3: Top Three Predictive Features by Pesticide Type Based on SHAP Values; Figure S3: SHAP Summary Plots of Top Predictors for 14-Day Mortality: LASSO-Only vs. LASSO + Clinical Logistic Regression Models; Table S4: Feature Importance Based on Logistic Regression Coefficients and Odds Ratios in the Final LASSO Model; Table S5: Baseline Characteristics by Predicted Risk Groups; Table S6: Comparative Performance Metrics between the Final LASSO Model and Univariate Logistic Regression Using APACHE II Score; Figure S4: Distribution of APACHE II Score. The Python analysis code and a synthetic dataset with the same structure as the original patient data (randomly generated values for privacy) are available at our GitHub repository (https://github.com/5454dls/pesticide-poisoning-mortality-prediction (accessed on 15 October 2025)).

## Abbreviations

The following abbreviations are used in this manuscript:

ALT	Alanine transaminase
AKI	Acute renal injury
APACHE II	Acute Physiology and Chronic Health Evaluation II
AST	Aspartate transaminase
AUC	Area under the receiver operating characteristic curve
BE	Base excess
BP	Blood pressure
BUN	Blood urea nitrogen
CKD	Chronic kidney disease
Cl	Chloride
CM	Carbamate
DBP	Diastolic blood pressure
EMR	Electronic medical record
GCS	Glasgow Coma Scale
HCO_3_^−^	Bicarbonate
ICU	Intensive care unit
LASSO	Least absolute shrinkage and selection operator
LIME	Local interpretable model-agnostic explanation
LR	Logistic regression
MAP	Mean arterial pressure
OR	Odds ratio
PR	Pulse rate
PSS	Poisoning Severity Score
RDW	Red blood cell Distribution Width
PDW	Platelet Distribution Width
RF	Random forest
SAPS	Simplified Acute Physiology Score
SBP	Systolic blood pressure
SHAP	SHapley Additive exPlanations
SOFA	Sequential Organ Failure Assessment
UA	Uric acid
