# Supplementary material for: An In-Hospital Mortality Prediction Model for Acute Pesticide Poisoning in the Emergency Department
Source: Toxics. 2025 Oct 18;13(10):893. doi: 10.3390/toxics13100893 (PMC12567830; doi:10.3390/toxics13100893)
Supplement: Supplementary file 1 [file toxics-13-00893-s001.zip › toxics-3819981-supplementary.pdf]

toxics

MDPI

Supplementary Table S1. Baseline Characteristics.

Variable	All_Patients	Train_Set	Test_Set	p_Value
Number of patients	n = 1056	n = 739	n = 317	
Age, years	63.0 ± 15.3	62.9 ± 15.3	63.3 ± 15.3	0.74
Sex, male (%)	666 (63.1)	462 (62.5)	204 (64.4)	0.62
BMI, kg/m²	22.7 ± 3.1	22.7 ± 3.1	22.7 ± 3.1	0.75
Event (death=1) (%)	171 (16.2)	120 (16.2)	51 (16.1)	1.00
Alcohol history, yes (%)	483 (45.7)	343 (46.4)	140 (44.2)	0.55
Diabetes mellitus, present (%)	194 (18.4)	131 (17.7)	63 (19.9)	0.46
Hypertension, present (%)	394 (37.3)	267 (36.1)	127 (40.1)	0.25
Pulmonary disease, present (%)	92 (8.7)	67 (9.1)	25 (7.9)	0.61
Cardiovascular disease, present (%)	66 (6.2)	46 (6.2)	20 (6.3)	1.00
Chronic kidney disease, present (%)	13 (1.2)	8 (1.1)	5 (1.6)	0.55
Neuropsychiatric disease, present (%)	201 (19.0)	140 (18.9)	61 (19.2)	0.98
Current smoker (%)	338 (32.0)	248 (33.6)	90 (28.4)	0.12
Ex-smoker (%)	43 (4.1)	30 (4.1)	13 (4.1)	1.00
Systolic blood pressure, mmHg	132.2 ± 27.1	132.1 ± 27.0	132.5 ± 27.3	0.99
Diastolic blood pressure, mmHg	77.0 ± 14.4	77.0 ± 14.4	77.0 ± 14.4	0.97
Mean arterial pressure, mmHg	95.5 ± 17.2	95.3 ± 17.3	95.9 ± 16.8	0.75
Pulse rate, /min	87.8 ± 16.1	87.5 ± 16.5	88.5 ± 15.2	0.17
Respiratory rate, /min	19.0 ± 3.8	18.9 ± 3.9	19.0 ± 3.7	0.57
Body temperature, °C	36.3 ± 0.8	36.3 ± 0.8	36.3 ± 0.8	0.99
Glasgow Coma Scale, total	12.7 ± 3.6	12.7 ± 3.6	12.7 ± 3.6	0.76
Hemoglobin, g/dL	14.0 ± 1.8	14.0 ± 1.8	13.8 ± 1.9	0.08
White blood cell, /µL	12.0 ± 5.7	12.2 ± 5.6	11.8 ± 5.8	0.17
Platelet count, ×10³/µL	246.3 ± 70.9	247.0 ± 70.0	244.8 ± 73.1	0.34
Glucose, mg/dL	146.0 ± 53.3	147.9 ± 54.8	141.3 ± 49.4	0.11
Blood urea nitrogen, mg/dL	16.1 ± 6.9	16.2 ± 7.2	15.7 ± 6.2	0.76
Serum creatinine, mg/dL	1.0 ± 0.6	1.0 ± 0.6	1.0 ± 0.5	0.51
Aspartate aminotransferase, IU/L	35.9 ± 29.3	35.7 ± 29.1	36.2 ± 29.8	0.80
Alanine aminotransferase, IU/L	23.1 ± 16.6	23.5 ± 17.2	22.4 ± 15.0	0.36
Partial pressure of oxygen, mmHg	88.2 ± 27.0	87.5 ± 25.2	89.8 ± 30.7	0.99
Partial pressure of carbon dioxide, mmHg	36.6 ± 7.7	36.6 ± 7.6	36.6 ± 7.8	0.73
Arterial pH	7.4 ± 0.1	7.4 ± 0.1	7.4 ± 0.1	0.71
Bicarbonate, mmol/L	21.1 ± 5.0	20.9 ± 5.0	21.4 ± 5.0	0.18
Total bilirubin, mg/dL	0.6 ± 0.4	0.6 ± 0.4	0.6 ± 0.3	0.62
Serum albumin, g/dL	4.3 ± 0.5	4.3 ± 0.5	4.2 ± 0.5	0.01*
Serum sodium, mEq/L	141.6 ± 3.6	141.4 ± 3.6	142.0 ± 3.7	0.01*
Serum potassium, mEq/L	4.0 ± 0.6	4.0 ± 0.6	3.9 ± 0.6	0.70
Serum chloride, mEq/L	102.6 ± 4.4	102.4 ± 4.4	103.2 ± 4.3	0.01*
Anion gap, mEq/L	17.8 ± 6.0	18.0 ± 6.1	17.4 ± 5.9	0.13
Activated partial thromboplastin time, sec	28.3 ± 5.8	28.2 ± 5.9	28.5 ± 5.8	0.34
Prothrombin time INR	1.1 ± 0.1	1.1 ± 0.1	1.1 ± 0.1	0.95

C-reactive protein, log(mg/L)	1.2 ± 1.1	1.2 ± 1.1	1.3 ± 1.2	0.47
Creatine kinase, log(IU/L)	4.9 ± 0.7	4.9 ± 0.7	4.9 ± 0.7	0.78
CK-MB, log(IU/L)	2.4 ± 1.2	2.4 ± 1.2	2.5 ± 1.1	0.72
Serum lactate, mmol/L	2.70 (1.50, 4.80)	2.70 (1.50, 4.80)	2.80 (1.60, 4.70)	0.54
Pesticide category				
Glufosinate	228 (21.6)	160 (21.7)	68 (21.5)	1.00
Glyphosate	234 (22.2)	164 (22.2)	70 (22.1)	1.00
Organophosphate	111 (10.5)	78 (10.6)	33 (10.4)	1.00
Carbamate	15 (1.4)	10 (1.4)	5 (1.6)	1.00
Pyrethroid	85 (8.0)	59 (8.0)	26 (8.2)	1.00
Paraquat	133 (12.6)	93 (12.6)	40 (12.6)	1.00
Other pesticides	250 (23.7)	175 (23.7)	75 (23.7)	1.00
Amount of ingestion				
50-100mL	203 (19.2)	149 (20.2)	54 (17.0)	1.00
100-200mL	188 (17.8)	134 (18.1)	54 (17.0)	1.00
200-300mL	164 (15.5)	108 (14.6)	56 (17.7)	1.00
>300mL	150 (14.2)	101 (13.7)	49 (15.5)	1.00
Unknown	135 (12.8)	102 (13.8)	33 (10.4)	1.00

Data are presented as mean ± standard deviation, median (interquartile range), or count (%) as appropriate. P-values indicate comparisons between the training and test sets. Other pesticides include acetanilide, acetylaniline, alryoxylcarboxide, amide, anilin, arsenic, (aryloxy) phenopropionate, benzohydrazide, benzoate, chlorfenapyr, chloroacetamide, chloronicotinyl, diamide, diazine, dinitroaniline, endosulfan, fungicide, insect growth regulator, lambda cyhalothrin, neonicotinoid, niacin, oxadiazole, phenoxy, pyrol, sulfonylurea, sulfoximine, sulfuryl fluoride, tetramic acid, tetrazolium oxide, urea, and unknown pesticides. BMI, body mass index; BP, blood pressure; PR, pulse rate; RR, respiratory rate; BUN, blood urea nitrogen; HCO_3^-, bicarbonate.

Supplementary Table S2. Feature List; Full Cox, Clinical, LASSO, RF List.

Feature list name	Selected features
**Full Cox (HR p<0.05)**	PTINR_scaled, pH_scaled, Gluc_scaled, pCO2_scaled, MAP_adm, BT_adm, DBP_adm, age, MCHC, Cr, WBC, BUN, GCS_total_est, lactate_ER, phos, AST, PTsec, AG, BE, HCO_3^-, Kal, alcohol_bin, CKMB_log, Uprot_num, pest_paraquat, UWBC_cat, troponinT_bin, smoking_current, Uery_num, URBC_cat, Uglc_num, O2sat, RDW, pest_etc, Uleuko_num, Cl, pest_glyphosate, SBP_adm, UA, Alb, PR_adm, CRP1_log, dose_unknown, ALP, ethanol_log, MCV, PDW, smoking_ex, pest_pyrethroid, Ca, MPV, CK_log, ALT
**Clinical**	AG, ALT, AST, BT_adm, BUN, CKD_hx_bin, Cr, DM_bin, GCS_total_est, HCO_3^-, HTN_bin, Hct, Kal, MAP_adm, Na, PR_adm, RR_adm, T_bil, WBC, age, cardio_dz_bin, pH_scaled, pO2_scaled, pulmo_dz_bin, seizure_bin
**LASSO**	pest_paraquat, BE, GCS_total_est, alcohol_bin, PTINR_scaled, PDW, Cr, lactate_ER, Uleuko_num, age, HCO_3^-, Cl, ALP, Uery_num, RDW, Gluc_scaled, BUN, PR_adm, DBP_adm, Kal, O2sat, MCHC
**RF**	pest_paraquat, HCO_3^-, lactate_ER, BE, AG, Cr, pH_scaled, WBC, Gluc_scaled, GCS_total_est, age, phos, CRP1_log, BUN, AST, PDW, ALP, PR_adm, PTINR_scaled, RDW, pCO2_scaled, UA, PTsec, MCHC, CKMB_log, CK_log, Alb, BT_adm, MCV, Kal, Ca, ethanol_log, Uery_num, O2sat, MPV, MAP_adm

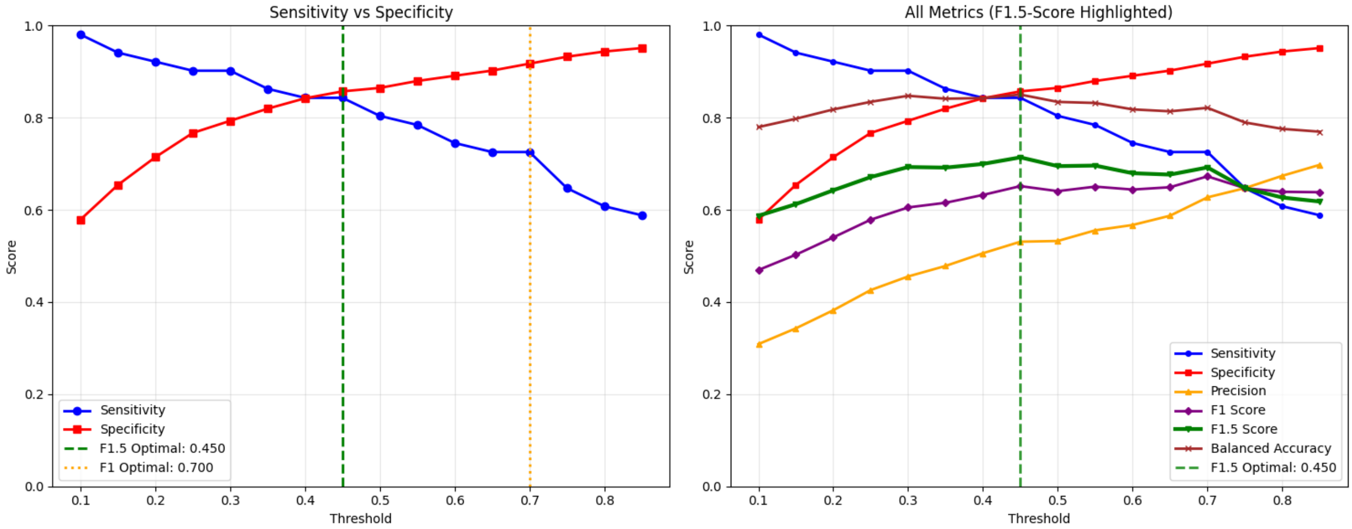

Supplementary Figure S1. Model Performance Metrics Across Thresholds.

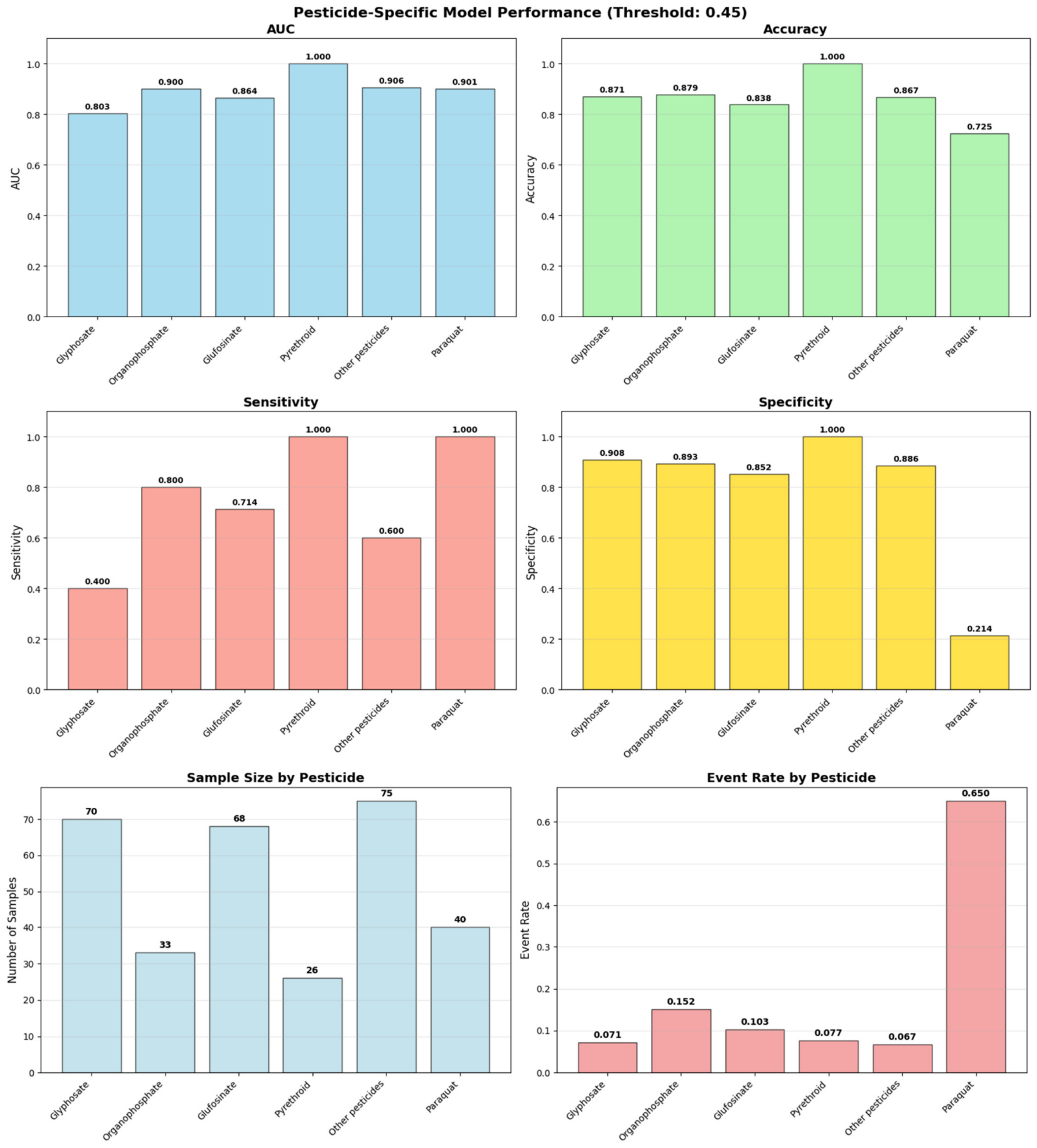

Supplementary Figure S2. Performance Metrics of the Pesticide-specific 14-day Mortality Prediction Models at a Threshold of 0.45. For each pesticide category (organophosphate, glufosinate, glyphosate, paraquat, and other pesticides), model performance was evaluated using AUC, accuracy, sensitivity, and specificity. Sample size and event rate for each group are also presented.

Supplementary Table S3. Top Three Predictive Features by Pesticide Type Based on SHAP Values.

Pesticide Type	1st Feature	2nd Feature	3rd Feature
**Paraquat**	Cr (0.888)	HCO_3^- (0.713)	Lactate_ER (0.684)
**Glyphosate**	HCO_3^- (0.552)	Lactate_ER (0.352)	Cr (0.323)
**Glufosinate**	HCO_3^- (0.438)	Cr (0.304)	GCS_total_est (0.301)
**Organophosphate**	GCS_total_est (0.455)	HCO_3^- (0.436)	Cr (0.387)
**Pyrethroid**	HCO_3^- (0.427)	Lactate_ER (0.326)	Cr (0.318)
**Other pesticides**	HCO_3^- (0.436)	Cr (0.321)	Lactate_ER (0.320)

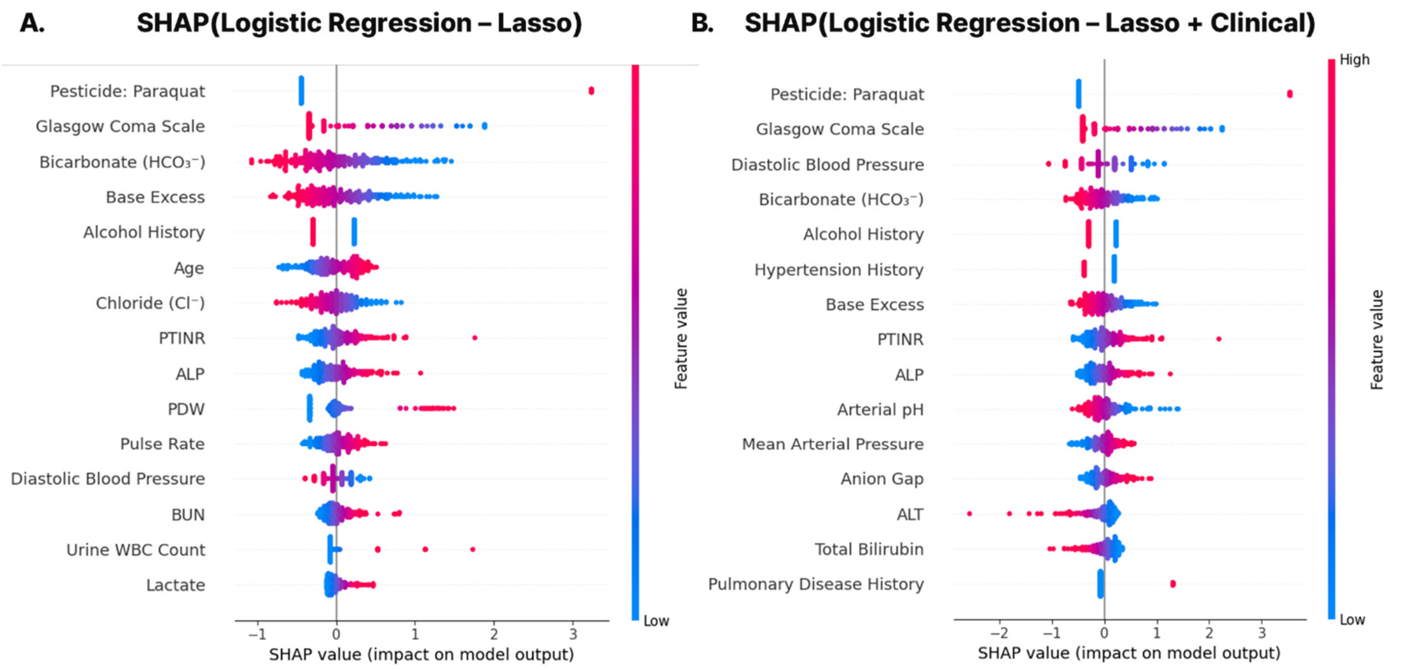

Supplementary Figure S3. SHAP Summary Plots of Top Predictors for 14-Day Mortality: LASSO-Only vs. LASSO + Clinical Logistic Regression Models.

Supplementary Table S4. Feature Importance Based on Logistic Regression Coefficients and Odds Ratios in the Final LASSO Model.

Rank	Feature	Coefficient (β)	Odds Ratio (OR)
**1**	pest_paraquat	+3.678	39.553
**2**	GCS_total_est	–0.664	0.515
**3**	HCO_3^-	–0.531	0.588
**4**	alcohol_bin	–0.520	0.594
**5**	BE	–0.445	0.641
**6**	PDW	+0.377	1.458
**7**	PTINR_scaled	+0.291	1.337
**8**	Cl	–0.277	0.758
**9**	Age	+0.273	1.315
**10**	Uleuko_num	+0.262	1.300
**11**	ALP	+0.242	1.274
**12**	PR_adm	+0.198	1.219
**13**	DBP_adm	–0.167	0.846
**14**	BUN	+0.154	1.166
**15**	lactate_ER	+0.131	1.140

Positive coefficients and OR >1 indicate increased mortality risk (risk factors), while negative coefficients and OR <1 indicate protective effects. Features are ranked by absolute magnitude of coefficient values.

Supplementary Table S5. Baseline Characteristics by Predicted Risk Groups.

Variable	All Patients	Low Risk	Medium Risk	High Risk	p-Value
Number of patients	n = 317	n = 105	n = 107	n = 105	
Predicted probability, median (IQR)	0.11 (0.03, 0.45)	0.02 (0.01, 0.03)	0.11 (0.06, 0.17)	0.73 (0.46, 0.94)	< 0.001*
Actual mortality (%)	51 (16.1)	0 (0.0)	5 (4.7)	46 (43.8)	< 0.001*
Age, yrs	63.3 ± 15.3	53.9 ± 13.3	65.9 ± 15.3	70.0 ± 12.5	< 0.001*
Sex, male (%)	204 (64.4)	69 (65.7)	67 (62.6)	68 (64.8)	0.89
BMI, kg/m²	22.7 ± 3.1	22.9 ± 3.2	22.7 ± 2.9	22.4 ± 3.2	0.397
APACHE II score	10.2 ± 6.8	5.3 ± 2.9	9.4 ± 4.3	16.0 ± 7.2	< 0.001*
Pesticide type, n (%)					< 0.001*
Glufosinate	68 (21.5)	20 (19.0)	28 (26.2)	20 (19.0)	
Glyphosate	70 (22.1)	27 (25.7)	31 (29.0)	12 (11.4)	
Organophosphate	33 (10.4)	10 (9.5)	12 (11.2)	11 (10.5)	
Carbamate	5 (1.6)	1 (1.0)	2 (1.9)	2 (1.9)	
Pyrethroid	26 (8.2)	10 (9.5)	12 (11.2)	4 (3.8)	
Paraquat	40 (12.6)	0 (0.0)	1 (0.9)	39 (37.1)	
Other pesticides	75 (23.7)	37 (35.2)	21 (19.6)	17 (16.2)	
Alcohol history, yes (%)	140 (44.2)	64 (61.0)	37 (34.6)	39 (37.1)	< 0.001*
Diabetes mellitus, present (%)	63 (19.9)	18 (17.1)	21 (19.6)	24 (22.9)	0.582
Hypertension, present (%)	127 (40.1)	31 (29.5)	46 (43.0)	50 (47.6)	0.021*
Pulmonary disease, present (%)	25 (7.9)	5 (4.8)	12 (11.2)	8 (7.6)	0.217
Cardiovascular disease, present (%)	20 (6.3)	4 (3.8)	6 (5.6)	10 (9.5)	0.219
CKD, present (%)	5 (1.6)	0 (0.0)	1 (0.9)	4 (3.8)	0.069
Neuropsychiatric disease, present (%)	61 (19.2)	22 (21.0)	24 (22.4)	15 (14.3)	0.278
Current smoker (%)	90 (28.4)	41 (39.0)	29 (27.1)	20 (19.0)	0.005*
Ex-smoker (%)	13 (4.1)	6 (5.7)	2 (1.9)	5 (4.8)	0.338
SBP, mmHg	132.5 ± 27.3	138.5 ± 22.7	133.3 ± 25.8	125.7 ± 31.6	0.008*
DBP, mmHg	77.0 ± 14.4	81.7 ± 12.3	77.4 ± 14.1	72.1 ± 15.3	< 0.001*
MAP, mmHg	95.9 ± 16.8	100.3 ± 13.9	95.9 ± 16.7	91.3 ± 18.4	0.002*
PR, /min	88.5 ± 15.2	84.3 ± 13.6	90.4 ± 14.0	90.7 ± 17.0	0.003*
RR, /min	19.0 ± 3.7	19.4 ± 1.8	19.1 ± 3.3	18.5 ± 5.3	0.858
BT, °C	36.3 ± 0.8	36.5 ± 0.5	36.4 ± 0.7	36.0 ± 0.9	< 0.001*
GCS	12.7 ± 3.6	14.7 ± 0.7	13.1 ± 2.8	10.5 ± 4.6	< 0.001*
Hb, g/dL	13.8 ± 1.9	14.1 ± 1.7	13.8 ± 1.9	13.5 ± 2.0	0.066
WBC, /μL	11.8 ± 5.8	8.6 ± 3.4	12.6 ± 5.4	14.1 ± 6.6	< 0.001*
Platelet count, ×10³/μL	244.8 ± 73.1	240.3 ± 62.9	249.7 ± 75.3	244.2 ± 80.5	0.581
Glucose, mg/dL	141.3 ± 49.4	125.8 ± 39.0	132.0 ± 35.3	166.4 ± 60.4	< 0.001*
Blood urea nitrogen, mg/dL	15.7 ± 6.2	13.3 ± 5.0	16.0 ± 5.6	18.0 ± 6.9	< 0.001*
Serum creatinine, mg/dL	1.0 ± 0.5	0.8 ± 0.2	0.9 ± 0.4	1.3 ± 0.7	< 0.001*
AST, IU/L	36.2 ± 29.8	32.2 ± 24.6	31.4 ± 25.8	45.3 ± 36.1	< 0.001*
ALT, IU/L	22.4 ± 15.0	24.0 ± 12.8	20.7 ± 12.7	22.5 ± 18.7	0.016*
PaO2, mmHg	89.8 ± 30.7	86.5 ± 22.3	89.8 ± 36.3	93.2 ± 31.9	0.386
PaCO2, mmHg	36.6 ± 7.8	39.2 ± 6.4	37.6 ± 6.9	32.9 ± 8.6	< 0.001*
Arterial pH	7.4 ± 0.1	7.4 ± 0.1	7.4 ± 0.1	7.3 ± 0.1	< 0.001*
Bicarbonate, mmol/L	21.4 ± 5.0	24.6 ± 3.2	22.1 ± 3.2	17.3 ± 5.2	< 0.001*

Total bilirubin, mg/dL	0.6 ± 0.3	0.6 ± 0.3	0.5 ± 0.3	0.6 ± 0.4	0.711
Serum albumin, g/dL	4.2 ± 0.5	4.3 ± 0.4	4.2 ± 0.5	4.0 ± 0.5	< 0.001*
Serum sodium, mEq/L	142.0 ± 3.7	142.8 ± 3.0	141.5 ± 3.6	141.7 ± 4.2	0.034*
Serum potassium, mEq/L	3.9 ± 0.6	3.9 ± 0.4	4.0 ± 0.6	3.9 ± 0.9	0.227
Serum chloride, mEq/L	103.2 ± 4.3	104.5 ± 3.6	103.0 ± 4.3	101.9 ± 4.8	< 0.001*
Anion gap, mEq/L	17.4 ± 5.9	14.0 ± 3.4	16.4 ± 4.3	21.8 ± 6.6	< 0.001*
aPTT, sec	28.5 ± 5.8	27.8 ± 4.4	27.8 ± 5.1	29.8 ± 7.5	0.151
PT-INR	1.1 ± 0.1	1.0 ± 0.1	1.0 ± 0.1	1.1 ± 0.2	< 0.001*
CRP, log(mg/L)	1.3 ± 1.2	0.9 ± 0.9	1.3 ± 1.1	1.6 ± 1.4	0.002*
Creatine kinase, log(IU/L)	4.9 ± 0.7	4.8 ± 0.7	4.8 ± 0.6	4.9 ± 0.7	0.195
CK-MB, log(IU/L)	2.5 ± 1.1	2.3 ± 1.0	2.4 ± 1.1	2.7 ± 1.2	0.038*
Serum lactate, mmol/L	2.80 (1.60, 4.70)	2.00 (1.20, 3.00)	2.40 (1.50, 4.00)	5.60 (3.18, 8.72)	< 0.001*

Risk groups were defined by tertiles of predicted probability: low ≤ 0.039; medium 0.040–0.264; high ≥ 0.265. Statistically significant p-values (< 0.05) are indicated with an asterisk (*).

Supplementary Table S6. Comparative Performance Metrics between the Final LASSO Model and Univariate Logistic Regression Using APACHE II Score.

Metric	Final LASSO-based LR model	APACHE II-based LR model
AUC (95% CI)	0.9229 (0.884–0.955)	0.8350 (0.781–0.888)
Accuracy	0.8549	0.7729
Sensitivity	0.8431	0.7059
Specificity	0.8571	0.7857
Precision	0.5309	0.3871
F1 Score	0.6515	0.5000
Balanced Accuracy	0.8501	0.7458

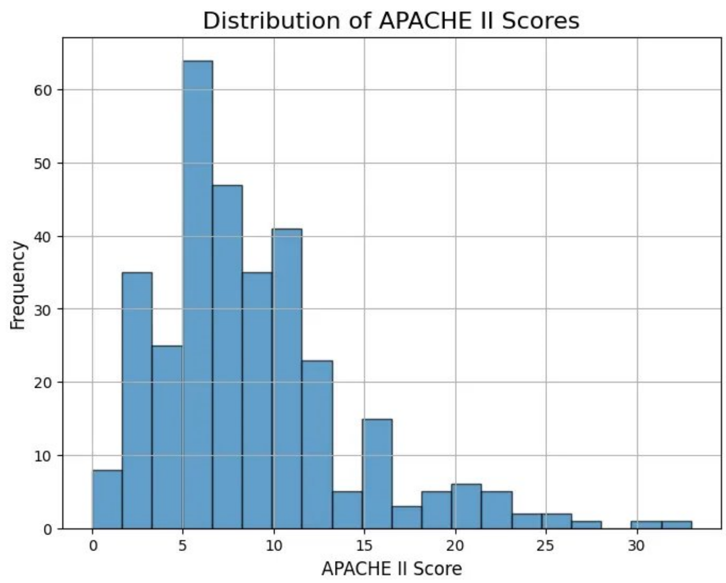

Supplementary Figure S4. Distribution of APACHE II score.
